# Non-Coding RNAs and Brain Tumors: Insights Into Their Roles in Apoptosis

**DOI:** 10.3389/fcell.2021.792185

**Published:** 2022-01-17

**Authors:** Omid Reza Tamtaji, Maryam Derakhshan, Fatemeh Zahra Rashidi Noshabad, Javad Razaviyan, Razie Hadavi, Hamed Jafarpour, Ameneh Jafari, Ali Rajabi, Michael R. Hamblin, Mahmood Khaksary Mahabady, Mohammad Taghizadieh, Hamed Mirzaei

**Affiliations:** ^1^ Students’ Scientific Research Center, Tehran University of Medical Sciences, Tehran, Iran; ^2^ Department of Pathology, Isfahan University of Medical Sciences, Isfahan, Iran; ^3^ Student Research Committee, Kashan University of Medical Sciences, Kashan, Iran; ^4^ Student Research Committee, School of Medicine, Shahid Beheshti University of Medical Sciences, Tehran, Iran; ^5^ Student Research Committee, School of Medicine, Mazandaran University of Medical Sciences, Sari, Iran; ^6^ Advanced Therapy Medicinal Product (ATMP) Department, Breast Cancer Research Center, Motamed Cancer Institute, ACECR, Tehran, Iran; ^7^ Proteomics Research Center, Shahid Beheshti University of Medical Sciences, Tehran, Iran; ^8^ School of Medicine, Kashan University of Medical Sciences, Kashan, Iran; ^9^ Laser Research Centre, Faculty of Health Science, University of Johannesburg, Johannesburg, South Africa; ^10^ Anatomical Sciences Research Center, Institute for Basic Sciences, Kashan University of Medical Sciences, Kashan, Iran; ^11^ Department of Pathology, School of Medicine, Center for Women’s Health Research Zahra, Tabriz University of Medical Sciences, Tabriz, Iran; ^12^ Research Center for Biochemistry and Nutrition in Metabolic Diseases, Institute for Basic Sciences, Kashan University of Medical Sciences, Kashan, Iran

**Keywords:** brain tumors, non-coding RNAs, apoptosis, microRNA, long non-coding (lnc) RNA

## Abstract

A major terrifying ailment afflicting the humans throughout the world is brain tumor, which causes a lot of mortality among pediatric and adult solid tumors. Several major barriers to the treatment and diagnosis of the brain tumors are the specific micro-environmental and cell-intrinsic features of neural tissues. Absence of the nutrients and hypoxia trigger the cells’ mortality in the core of the tumors of humans’ brains: however, type of the cells’ mortality, including apoptosis or necrosis, has been not found obviously. Current studies have emphasized the non-coding RNAs (ncRNAs) since their crucial impacts on carcinogenesis have been discovered. Several investigations suggest the essential contribution of such molecules in the development of brain tumors and the respective roles in apoptosis. Herein, we summarize the apoptosis-related non-coding RNAs in brain tumors.

## Introduction

Cancer is specified as uncontrolled cell growth and proliferation resulting in an imbalance between division and death of the cells as a result of many different factors including physicochemical or biological agents (Evan and Vousden, 2001; Jafari et al., 2021a). Today in the world, cancer is still one of the paramount health issues (Nikolaou et al., 2018; Jafari et al., 2020). Brain tumors are almost specified with great morbidity and mortality rate (Weller et al., 2015). Glioma is the most common and aggressive diagnosed tumor of the central nervous system (CNS) with low survival and high recurrence rate (Khani et al., 2019; Xu et al., 2020). Among all tumors, glioma represents just 1–2%, whereas approximately 30% of all preliminary and 80% of every malignant tumor of the brain are affiliated with glioma, which responsible for many brain disease-related deaths. It is thought neurological stem and/or progenitor cells are responsible for brain tumors. Based on morphological resemblance to the normal neuroglial cells, these heterogeneous tumors histologically are classified into oligodendrogliomas, astrocytomas, and ependymomas (Weller et al., 2015). Based on classification by WHO, there are two classes of glioma with four distinct grades including low- (I and II) and high- (III and IV) grades. Classification of glioma based upon molecular characteristics including genetics, gene expression, DNA methylation, and so on plays a pivotal role in diagnosis, prognosis, and treatment (Li et al., 2019a). Despite much research and clinical investigation in recent years, the prognosis and treatment of glioma are not so desirable (Xu et al., 2020). Great proliferation rate with high resistance to therapies, sensitivity of the CNS and low ability to repair itself, and inability of most drugs to pass the blood brain barrier (BBB) prevent impressive treatment of glioma (So et al., 2021). One of the malignant glioma’s hallmarks is its ability to infiltrate the brain, leads to avoidable recurrence post local therapy (So et al., 2021). Nowadays, existing therapeutic approaches include resection surgery, chemotherapy, radiotherapy, and combination therapies. However, contrary to considerable progress in treatment options, after initial resection, frequently quick progression and a high rate of recurrence are shown in patients with glioma (Aldape et al., 2019). Discovering new, innovative, and effective therapies for brain tumors depends on the improved perception of the molecular aspects of this disorder (Aldape et al., 2019).

Several studies have indicated the contribution of apoptosis in the pathophysiology of brain tumors (Mellai and Schiffer, 2007; Fernald and Kurokawa, 2013; Mohamed Yusoff, 2015). Apoptosis is a physiological process during aging and development which led to programmed cell death (Majtnerová and Roušar, 2018; Wang, 2020). The term of apoptosis was first used in a paper published in 1972 by Kerr, Wyllie and Currie to describe a hemostatic process which maintains cell populations by removing damaged and unnecessary cells (Majtnerová and Roušar, 2018). Apoptosis is controlled genetically and is an eventually conserved process among multicellular organisms (D’Arcy, 2019; Jafari et al., 2021b). The mediators of apoptosis are enzymes named caspases. Caspases are cytosolic proteases with the proteolytic activity and they are expressed as pro-enzymes in most cells. When a caspase is activated, it can also activate other procaspases and can induce a cascade of activated caspases which can finally led to cellular death (D’Arcy, 2019; Elmore, 2007). A wide range of conditions and stimuli can initiate apoptosis (Elmore, 2007; Majtnerová and Roušar, 2018). Today three main pathways are described in apoptosis including extrinsic, intrinsic and perforin/granzyme-mediated pathways (Majtnerová and Roušar, 2018). The intrinsic pathway is mitochondrial and initiate with death signals like DNA damage and withdrawal of trophic factors. The response to death signals is disruption of the mitochondrial membrane permeability which led to activation of a series of caspases (Wang, 2020). The extrinsic pathway is induced *via* death receptors placed on plasma membrane such as TNF and Fas receptors. This pathway also eventually leads to the activation of caspases (Wang, 2020). In perforin/granzyme pathway, apoptosis can be caused by each of the granzyme A or granzyme B. Both extrinsic and intrinsic pathway in addition to granzyme B pathway led to apoptosis via cleavage of caspase-3. But granzyme A pathway is initiated via single stranded DNA damage and induce apoptosis via a caspase independent pathway (Elmore, 2007). A variety of morphological and biochemical changes occurs in cells during apoptosis (Majtnerová and Roušar, 2018). Morphological changes contain cellular shrinkage, nuclear fragmentation and chromatin condensation. The cytoplasmic membrane undergoes changes including budding, bleeding, loss of integrity and transfer of phosphatidylserine (PS) to the extracellular part of membrane. The exposure of PS in outer membrane helps recognition and swallowing apoptotic cells by macrophages. These changes can be observed by light and electron microscopes but electron microscopy give more valuable information and show us more morphological changes (Elmore, 2007; Pistritto et al., 2016; Xu et al., 2019a). Some of the biochemical features which are observed in apoptotic cells contain protein cross-linking, DNA segmentation, and nuclear and cytoskeletal proteins cleavage. Apoptotic cells also form apoptotic bodies and express the ligands necessary for recognition by phagocytic cells and finally, they are identified and removed by phagocytic cells (Elmore, 2007).

**FIGURE 1:**
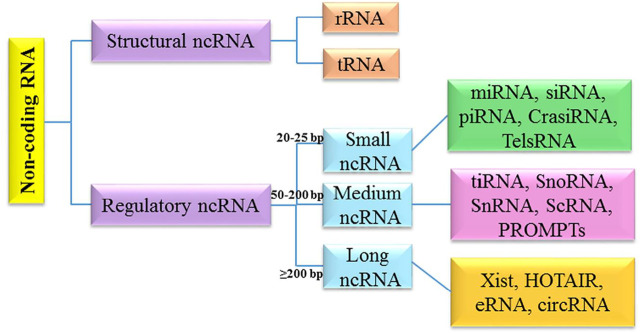
Classification of non-coding RNAs.

It has been shown that different cellular/molecular mechanisms are associated with apoptosis-related processes in brain tumors. Along with genetic mechanisms, epigenetic mechanisms (e.g., non-coding RNAs networks) greatly affect the regulation of apoptosis-related processes. The non-coding RNAs (ncRNAs) belong to a class of RNAs, which function at the RNA level (Nicoloso and Calin, 2008; Pop et al., 2018). NcRNAs are highly heterogeneous in length, structure and cellular function (Hashemian et al., 2020; Mirzaei and Hamblin, 2020; Balandeh et al., 2021; Dashti et al., 2021; Mahjoubin-Tehran et al., 2021). They can be classified into two main classes: structural non-coding RNA and regulatory non-coding RNA. Structural ncRNA include ribosomal RNA (rRNA) and transfer RNA (tRNA). Regulatory ncRNAs are divided into three subclasses based on the length, and include different types from small nucleolar/nuclear RNA (snoRNA/snRNA), small interfering RNAs (siRNA), microRNA (miRNA), and piwiRNA (PiRNA) to Xist and circRNA (Zhang et al., 2019a) (Figure 1).

In this study, we describe the structure and biogenesis of miRNA, lncRNA, and circRNA, then summarize the apoptosis-related non-coding RNAs in brain tumors.

## Non-Coding RNAs and Brain Tumors

### MicroRNAs Biogenesis

MiRNAs are small single stranded non-coding RNAs, with the mean length of nearly 22 nucleotides, which play important roles in regulating gene expression at the post-transcriptional level and RNA silencing (Shabaninejad et al., 2019). In 1993, lin-4, the discovery of the first miRNA, lin-4, by the Ambros and Ruvkun team led to a revolution in molecular biology (Lee et al., 1993; Achkar et al., 2016; Gebert and MacRae, 2019; O'Brien et al., 2018).

Generally, miRNA is critical for normal animal development and is capable of regulating a variety of biological processes and signaling pathways like metabolism and differentiation as well as rapid growth or proliferation. Increasing evidence have shown the correlation between miRNA deregulation and numerous kinds of human diseases, including heart disease, metabolic disorder, and cancer. In addition, miRNAs contribute importantly to invasion, metastasis and the tumor angiogenesis and are capable of exiting from the cells through vesicles and entering the extra-cellular fluids (O'Brien et al., 2018; Lin and Gregory, 2015).

RNA polymerase II (Pol. II) enzyme begins miRNAs biogenesis in the nucleus and approximately half of the miRNAs have been identified to be intragenic, largely derived from the introns, whereas the others to be intergenic with their specific promoters (Gebert and MacRae, 2019).

Therefore, a long transcript referred to as the primary microRNA (pri-miRNA) is generated after the transcription by Pol. II and hence Pri-miRNA may create a cluster of two or more miRNAs or a miRNA. Furthermore, it has a local stem loop structure, undergoing several steps of maturation like splicing, polyadenylation, as well as capping. Scission of Pri-miRNA to pre-miRNA is conducted by a micro-processor complex which has two parts: the double-strand RNase DROSHA and its crucial cofactor; that is, DiGeorge syndrome critical region 8 (DGCR8) (Lee et al., 1993; Ha and Kim, 2014; Lin and Gregory, 2015; Gebert and MacRae, 2019).

**FIGURE 2:**
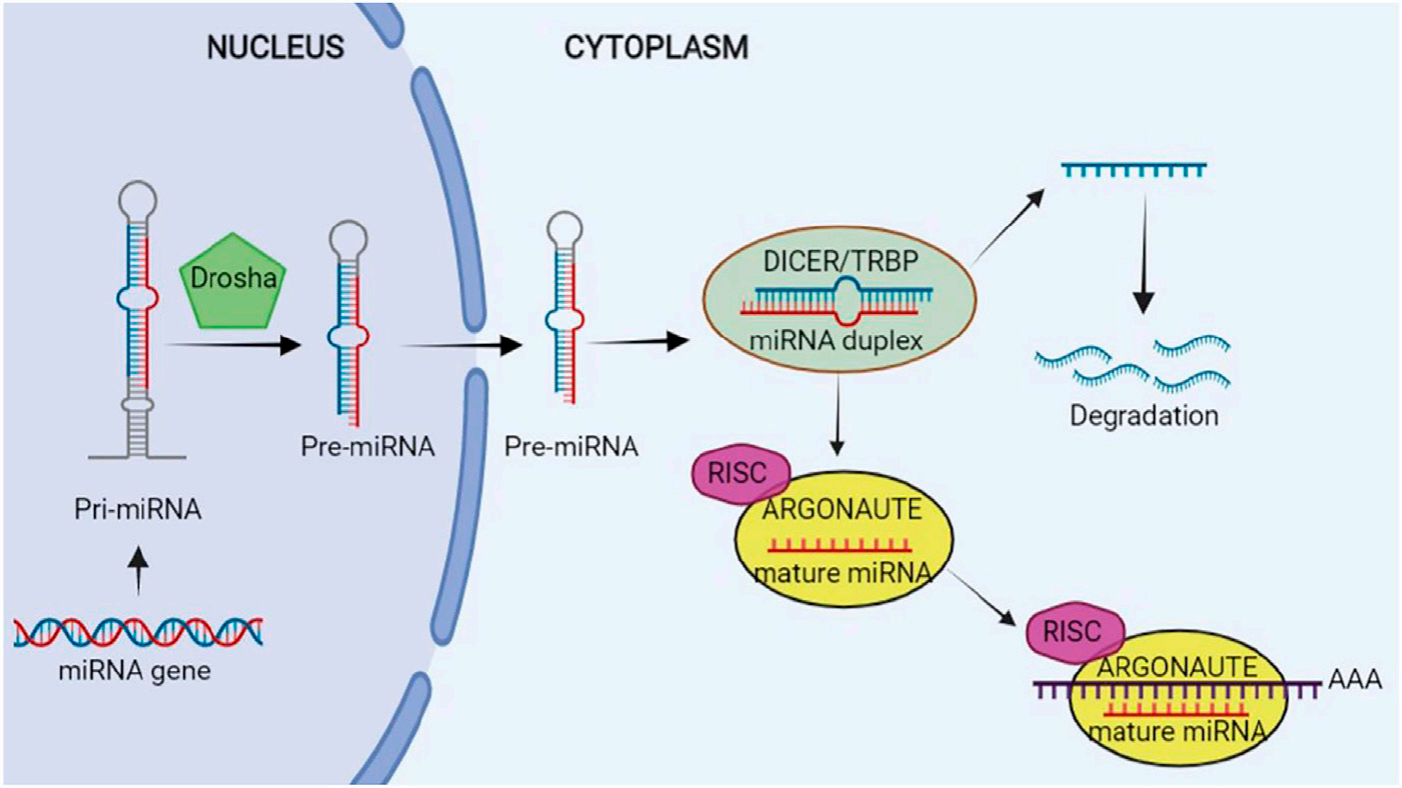
Biogenesis of microRNA (miRNA). This figure is created by www.BioRender.com.

Research has shown a length of nearly 65 nucleotides of pre-miRNAs with a hairpin-like structure that is the same as the pri-miRNA. Then, Exportin 5 translocates pre-miRNA into cytoplasm, wherein its maturity ends. Pre-miRNA is converted to a mature ∼22 nucleotide miRNA duplex by RNase III DICER1 enzyme and its binding protein, and transactivation response element RNA-binding protein (TRBP) in the cytoplasm. A strand works as one of the guide miRNAs and the other is loaded to Argonaute (AGO) for making a complex: RNA-induced silencing complex (RISC). The complex is assembled by two steps of loading and unwinding of RNA duplex. RISC is capable of binding to the 3′-UTR of the target mRNAs from 5′-UTR of miRNA that may repress translation or degrade mRNA (Ha and Kim, 2014; Lin and Gregory, 2015). The schematic steps of biogenesis of miRNA is illustrated in Figure 2.

MiRNAs may target and regulate a majority of the protein-coding genes due to the existence of one conserved binding site for miRNAs in the above genes. Additionally, numerous mRNAs may be targeted by a miRNA (Ha and Kim, 2014).

### MicroRNAs and Apoptosis in Brain Tumors

The miRNA-342 is elevated in glioblastoma cell, and could directly modulate BCL2 expression, suggesting a possible role in apoptosis induction (Ghaemi et al., 2020). The miRNA-16 can promote apoptosis by targeting BCL2 in glioblastoma cell (Yang et al., 2014). The miR-148a is elevated in glioblastoma cell. miR-148a inhibition could induce apoptosis *via* pro-apoptotic molecule BIM in glioblastoma (Kim et al., 2014). The elevated level of miR-330-5p in glioblastoma could induce cell apoptosis *via* targeting ITGA5 expression in glioblastoma cells (Feng et al., 2017). The miR-758-5p was up-regulated in glioblastoma. It can promote apoptosis by decreasing ZBTB20 in glioblastoma cell (Liu et al., 2018a). There is a downregulation of miR-543 in glioblastoma and its upregulation can induce apoptosis by targeting ADAM9 (Ji et al., 2017). MiR-152-3p affected the apoptosis of glioblastoma cells via NF2 overexpression (Sun et al., 2017). The inhibition of increased miR-210 in glioblastoma can increase ROD1 expression and apoptosis (Zhang et al., 2015). The overexpression of miR-125a-3p leads to decreased Nrg1 and increased apoptosis in glioblastoma cell (Yin et al., 2015). In another study, miR-500a-5p inhibited apoptosis in glioblastoma through targeting CHD5 mRNA 3′-UTR (Liu et al., 2018b). MiR-146a was down regulated in glioblastoma. In addition, upregulation of miR-146a suppresses Notch1 and stimulate in glioblastoma (Hu et al., 2016) MiR-125b was markedly up-regulated in glioma. MiR-125b regulates cell apoptosis by different signaling pathways (Wu et al., 2013). Elevated levels of miR-374b are reported in glioma. MiR-374b is implicated in the glioma progression by regulating apoptosis *via* targeting GATA3 and SEMA3B (Gao et al., 2019a). Upregulation of decreased miR-378a-3p in glioblastoma decreased the expression of TSPAN17 and increased apoptosis (Guo et al., 2019). MiR-152-3p induced glioma cell apoptosis *via* decreasing DNMT1 (Sun et al., 2017). In astrocytoma tumor cells, miRNA-124-3p was up-regulated and its over-expression leads to increasing apoptosis via targeting 3′-UTR of PIM1 (Deng et al., 2016). Furthermore, the over-expression of miRNA-160a-5p is able to enhance apoptosis in astrocytoma cells *via* decreasing Fas-activated serine/threonine kinase (FASTK), an anti-apoptotic agent (Zhi et al., 2013). The induction of an increase in MiR-181b-5p level in astrocytoma showed an increase in apoptosis through targeting NOVA1 in astrocytoma (Zhi et al., 2014). A study revealed a significant down-regulation of miR-106a-5p in astrocytoma. The Over-expression of miR-106a-5p leads to decrease in FASTK expression and increase in apoptosis (Zhi et al., 2013). Besides, miRNA-10b was up-regulated in medulloblastoma cell. miRNA-10b inhibition could also decrease the expression of BCL2 and MCL-1 protein expression in medulloblastoma cell lines (Pal and Greene, 2015). Xu et al. (2014) found that over-expression of miR-22 stimulated apoptosis *via* inhibiting PAPST1 in medulloblastoma cell. MiR-378 was down-regulated in medulloblastoma. The over-expression of miR-378 showed promoting apoptosis through targeting UHRF1 in medulloblastoma (Zhang et al., 2017). MiR-383 negatively regulated PRDX3. The over expression of miR-383 and inhibiting PRDX3 could leads to stimulating apoptosis in medulloblastoma (Li et al., 2013).

Meningioma cell lines show a low level miRNA-34a-3p and miRNA-34a-3p upregulation can stimulate cancer cell apoptosis *via* decreasing BCL2 protein (Werner et al., 2017). MiR-29c-3p was up-regulated in meningioma cell. MiR-29c-3p induces apoptosis via targeting PTX3 (Dalan et al., 2017). Apoptosis-related miRNAs in brain tumors and their expression were summarized in Table 1.

**TABLE 1:** Apoptosis-related miRNAs in brain tumors.

	Genomic coordinates	Brain tumors	miRNAs	Targets	Model	Type of cell line	Ref
Up regulated miRNA expression	q32.2 chr1:207801852–207801939 (-)	Meningioma	miRNA-29c-3p	PTX3	*In vitro*	Human tissues and meningioma cell line MEN-117 and MEN-141	Dalan et al. (2017)
					*In vivo*		
	q31.1 chr2:176150303–176150412 (+)	Medulloblastoma	miRNA-10b	Bcl2-MCL1	*In vitro*	medulloblastoma cell lines DAOY and UW228 and human sample	Pal and Greene, (2015)
	p15.2 chr7:25949919–25949986 (-)	Glioblastoma	miRNA-148a	BIM	*In vitro*	GBM cell lines U87, U373, A172, T98G, SNB-19 and U251	Kim et al. (2014)
					*In vivo*		
	p23.1 chr8:9903388–9903472 (-)	Astrocytoma	Mirna-124-3p	PIM1	*In vitro*	Human astrocytoma cell line u251 and human tissues	Deng et al. (2016)
	p15.5 chr11:568089–568198 (-)	Glioblastoma	miRNA-210	ROD1	*In vitro*	Tumor tissues and GBM cell line U87MG, U251	Zhang et al. (2015)
	q24.1 chr11:122099757–122099844 (-)	Glioblastoma	miRNA-125b	P53	*In vitro*	U251 and U87 cells, rat GMB C6 cells and human brain tissues	Wu et al. (2013)
				P38MAPK			
	q32.2 chr14:100109655–100109753 (+)	Gliomoblastoma	miRNA-342	Bcl2	*In vitro*	Glioblastoma cell line U251 and U87	Ghaemi et al. (2020)
	p11.23 chrX:50008431–50008514 (+)	Glioblastoma	miRNA-500a-5p	CHD5	*In vitro*	Tumor tissues sample	Liu et al. (2018b)
					*In vivo*	Glioblastoma cell lines U-87MG, U251	
	q13.2 chrX:74218547–74218618 (-)	Glioblastoma	miRNA-374b	GATA3	*In vitro*	Glioma cell line U251 and human tissues sample	Gao et al. (2019a)
				SEMA3B			
Down regulated miRNA expression	p36.22 chr1:9151668–9151777 (-)	Meningioma	Mirna-34a-3p	Bcl2	*In vitro*	Ben-Men-1	Werner et al. (2017)
	q32.1 chr1:198858873–198858982 (-)	Astrocytoma	miR-181b-5p	NOVA1	*In vitro*	Human tissues and astrocytoma cell lines U251 and U87	Zhi et al. (2014)
	q32 chr5:149732825–149732890 (+)	Glioblastoma	miRNA-378a-3p	TSPAN17		GBM cell lines U87MG and MT-330 and tumor tissues	Guo et al. (2019)
	q32 chr5:149732825–149732890 (+)	Medulloblastoma	miRNA-378	UHRF1	*In vivo*	DAOY and HEK 293T cell line	Zhang et al. (2017)
					*In vitro*	Tumor tissues	
	q33.3 chr5:160485352–160485450 (+)	Glioblastoma	miRNA-146a	Notch1	*In vitro*	GBM tissues, U87, U251, A172	Hu et al. (2016)
	p22 chr8:14853438–14853510 (-)	Medulloblastoma	miRNA-383	PRDX3	*In vitro*	Human sample and cell line DAOY, D283 and D341	Li et al. (2013)
	q14.2 chr13:50048973–50049061 (-)	Glioblastoma	miRNA-16	BCL2	*In vitro* and *in vivo*	Glioblastoma cell line U87 and U 373 and human sample	Yang et al. (2014)
	q32.31 chr14:101026020–101026107 (+)	Glioblastoma	miRNA-758-5p	ZBTB20	*In vivo*	Human tissues and U118, LN-299, H4, A172, U87-MG, and U251	Liu et al. (2018a)
	q32.31 chr14:101031987–101032064 (+)	Glioblastoma	miRNA-543	ADAM9	*In vitro*, human	U87, U251, LN229, and T98, human tissues	Ji et al. (2017)
	p13.3 chr17:1713903–1713987 (-)	Medulloblastoma	miRNA-22	PAPST1	*In vivo*	medulloblastoma cell lines D341 and DAOY and tumor tissues	Xu et al. (2014)
					*In vitro*		
	q21.32 chr17:48037161–48037247 (-)	Glioblastoma	MiRNA-152-3P	DVMT1	*In vitro*	U251, U87, T98-G and A172	Sun et al. (2017)
				NF2			
	q13.32 chr19:45638994–45639087 (-)	Glioblastoma	miRNA-330-5p	ITGA5	*In vitro*	U87, U251, and U373	Feng et al. (2017)
	q13.41 chr19:51693254–51693339 (+)	Glioblastoma	miRNA-125a-3p	Nrg1	*In vitro*	Animal sample	Yin et al. (2015)
					*In vivo*	Tumor sample	
						Glioblastoma cell line U251/U87-MG	
	q26.2 chrX:134170198–134170278 (-)	Astrocytoma	MiRNA-106a-5p	FASTK	*In vitro*	Astrocytoma cell line U251 and human tissues samples	Zhi et al. (2013)

### Biogenesis of lncRNAs

NcRNAs ability to regulate gene expression at transcriptional and post-transcriptional levels explains their house-keeping functions in numerous biological processes (Statello et al., 2021). Overall, researchers have described lncRNAs as long RNA transcripts of ≥200 nucleotides, which do not result in protein production (Nam et al., 2016). Moreover, they contribute importantly to the adjustment of the translation machineries and in its modulation *via* regulating the essential performance of other ncRNAs like small nucleolar RNA (snoRNA), miRNAs, and so forth. Several scholars presented many regulatory patterns that are under the control of numerous lncRNAs influencing different cellular activities that are correlated to the normal development and pathophysiology of some diseases like different kinds of cancers, neurological and cardio-vascular conditions, as well as metabolic and immunological dysfunctions (Maass et al., 2014). Therefore, it is important to know lncRNAs biogenesis for its differentiation from other kinds of RNA and deciphering its applicable prominence. Studies have also demonstrated transcription of numerous groups of lncRNAs from numerous DNA elements like enhancers, intergenic regions, and promoters in the eukaryotic genomes (Maass et al., 2014; Fang and Fullwood, 2016). In addition, they revealed the contribution of several mechanisms to the biogenesis of lncRNA like cleavage through ribonuclease P (RNaseP) for generating mature ends and forming protein (snoRNP) complex caps at their ends, snoRNA, and circular structures. However, there are not enough information of the action of synthesis and modulation of various lncRNAs (Dahariya et al., 2019).

Besides the dimension of the other classes of ncRNAs (e.g., siRNAs, miRNAs, and small sno/sn RNAs), lncRNAs enjoy the secondary and 3D structures that empower them for having protein-like and RNA functions (Cabili et al., 2015). Several studies determined the location of lncRNAs in the nucleus (Derrien et al., 2012), with the contribution of many lncRNAs to the cytoplasm (Cabili et al., 2015). In addition, it is possible for many lncRNAs to be transmitted to the near cells or serum via exosome trafficking. Earlier researchers have regarded lncRNAs as the by-products of transcription process. Nonetheless, lncRNAs contribute to the cell differentiation process, growth as well as pathogenesis of numerous illnesses like cancers (Maass et al., 2014). LncRNAs can modulate gene expression at the time of transcription, post-transcription, and even epigenetically (Cabili et al., 2015). Actually, lncRNAs greatly affect gene expression by modifying chromatin and remodeling, modifying histone, as well as changing the nucleosome localization. In comparison to the mRNAs, numerous lncRNAs are situated at the nucleus and researchers have shown the existence of fewer exons in the lncRNA genes in comparison to the mRNAs. Results have demonstrated the less effective splicing of lncRNAs than the mRNAs (Guo et al., 2020; Statello et al., 2021). In fact, they exhibited longer distances between and the branch point and 3′ splice site weaker internal splicing signals. Besides processing and transcription, lncRNAs frequently consist of the embedded sequence motifs with the ability of recruiting specific nuclear factors that enhance the lncRNA nuclear localization and function. Furthermore, researchers observed export of numerous lncRNAs to the cytosol with the similar export and processing pathways to the mRNAs. When the lncRNAs reached the cytoplasm, they experience particular arrangement processes for assigning various lncRNAs to the certain organelles or distribution in the cytoplasm for associating with several RNA-binding proteins (RBPs) (Statello et al., 2021). According to estimations, 50% of the pools of 70% of cytoplasmic lncRNAs occur in polysome fraction and lncRNAs at multiple level regulate the genes’ expression. (Carlevaro-Fita et al., 2016). In fact, through interactions with RNA, proteins, as well as DNA, lncRNAs may modulate the chromatin structure and functions and transcription of the distant and near genes, and influence the RNA splicing, translation, and stability. Research has showed the contribution of lncRNAs to the creation and modulation of nuclear condensates and organelles. They are capable of suppressing the genes’ expression via interferences in the transcription machinery and alter recruitment of the transcription factors or Pol II at the suppressed promoter (Pal and Greene, 2015) and histone modification (Pal and Greene, 2015; Lin et al., 2020) and declines the accessibility of chromatin (Statello et al., 2021).

**FIGURE 3:**
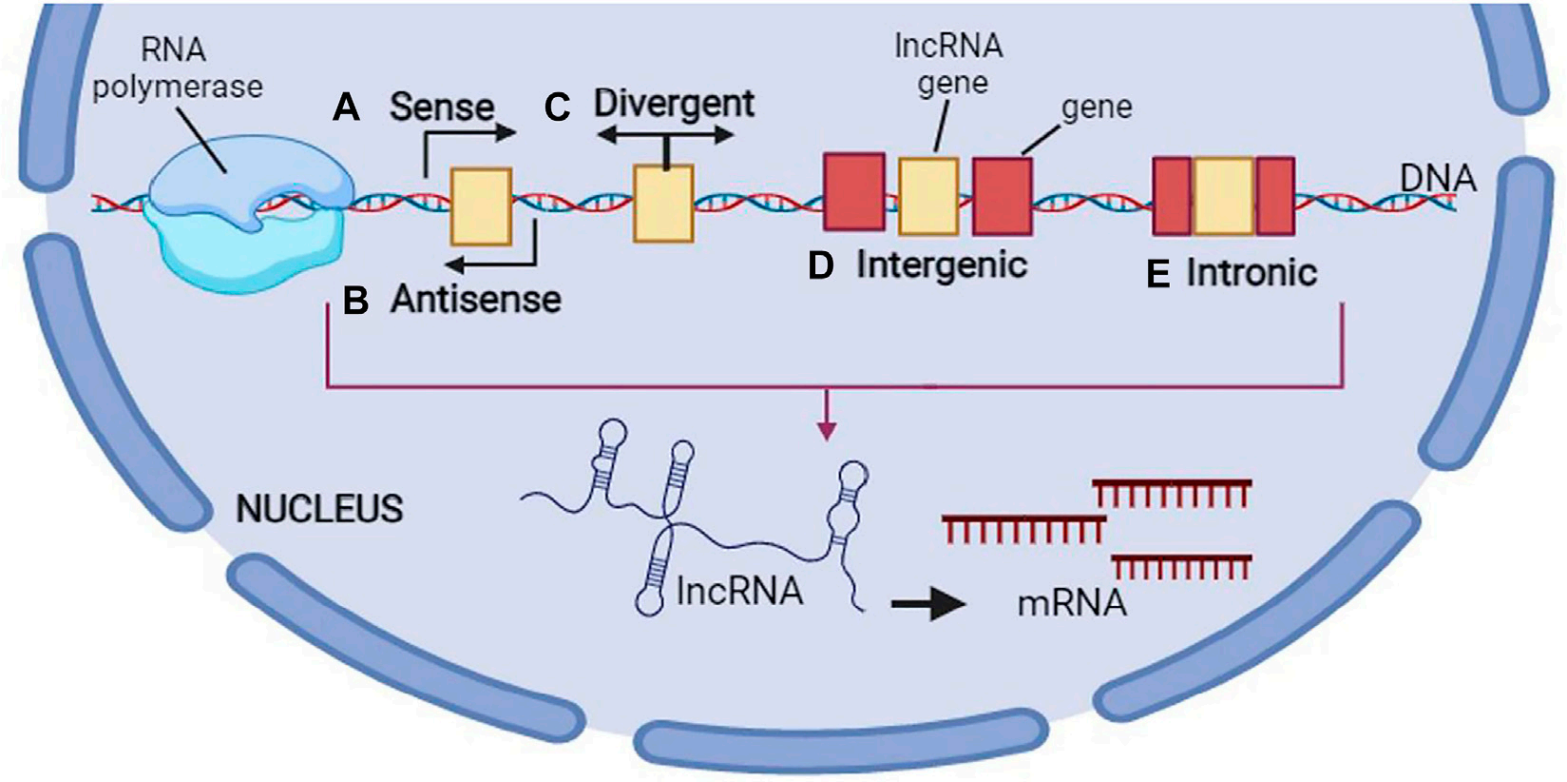
Biogenesis of long non-coding RNA (lncRNA). This figure is created by www.BioRender.com.

One of the other kinds of lncRNAs classification has been done according to their genomic profile or correlation with the protein coding genes: 1) one or more exons of a coding gene are overlapped by sense lncRNAs, 2) full or partial complementarity to transcripts on the opposite strand exhibits anti-sense transcripts, 3) an intron of a gene produces the intronic lncRNAs, 4) there is a similar promoter in both protein-coding genes and bi-directional transcripts, though their transcription is formed in the opposite direction, 5) inter-genic lncRNAs (lincRNAs) are situated between the protein-coding genes and their transcription occurs individually, 6) enhancer RNAs (eRNAs) have been found to be created from the enhancer regions of the protein-coding genes, and finally 7) the origin of circRNAs is the splicing processes of the protein coding genes that create the covalently-closed loops (Beermann et al., 2016). lncRNA biogenesis occurs in the nucleus resembling synthesizing the protein-coding transcripts. Moreover, histone modifications occur frequently for epigenetic marking of lncRNA promoters that are modulated by transcription factors, which advocate or hinder the gene expression (Guttman and Rinn, 2012). Like mRNAs, Pol II transcribes numerous lncRNAs whereas other lncRNA promoters protect the structures transcribed by Pol III. Therefore, there is a precise spatial or temporal regulation of lncRNA expression. Generally, there are fewer lncRNAs than mRNAs, though their expression is largely limited to the certain kinds of cells (Cabili et al., 2011; Derrien et al., 2012). Based on the transcriptome-wide investigations, lncRNAs exhibited more certain expression profiles than the mRNAs (Elmore, 2007; Nikolaou et al., 2018). In other words, their expression is done in a cell tissue-, type-, developmental stage or disease state-specific way. For reaching the mature forms, the nascent RNA transcripts experience several processing steps during and after transcription, like splicing, 5ʹ-capping, chemical base modification, as well as poly-adenylation. The schematic steps of biogenesis of lncRNA is shown in Figure 3 **.**

Researchers have found the biological contributions of lncRNAs as 1) the transcription regulators in cis or trans, 2) regulator of mRNA processing, protein activity, and posttranscriptional control, and 3) structuring the nuclear domains (Quinn and Chang, 2016). Overall, RNA polymerase II transcribe the lncRNAs, which exhibit hallmarks of the protein-coding genes, including conservation and chromatin structure of promoters, modulation of expression *via* morphogens and transcription factors, ranges of half-life tissue-specific expression, splicing, as well as other splice variants. Several lncRNAs enjoy a 3-helical structure at their 3′ end, created by cleavage via RnaseP, which helps protecting them against degradation (Yang et al., 2016).

### Long Non-coding RNAs and Apoptosis in Brain Tumors

LncRNA EGFR-AS1 expression was notably up-regulated in glioma. EGFR-AS1 inhibition induced apoptosis. MiR-133b is a target of EGFR-AS1. Lnc-EGFR-AS1 knockdown increased miR-133b expression. MiR-133b was able to decrease RACK1 expression. The over-expression of miR-133b causes glioma cells to increase their apoptosis by reducing N-cadherin, Vimentin, MMP-2 and Bcl-2 and elevating Bax, cleaved Caspase-3 and PARP expression (Dong et al., 2019). There is a remarkable increase in LncRNA HOXA11-AS in glioma. The direct target of LncRNA HOXA11-AS is miRNA-130-5p, i.e., it downregulates miRNA-130-5p. Apoptosis was greatly increased upon miR-130-5p transfection. The oncogenic function of LncRNA HOXA11-AS is partly related to miR-130a-5p-HMGB2 axis. The down-regulation of LncRNA HOXA11-AS leads to increasing apoptosis in glioma (Xu et al., 2019b). Zhang et al. (2020a) showed that glioma cells apoptosis was increased by LPP-AS2 inhibition. LPP-AS2 knockdown leads to decreased EGFR the expression. LPP-AS2 acts as a molecular sponge for miR-7-5p and inhibiting miR-7-5p reverses the cell apoptosis stimulated by LPP-AS2 knockdown. LPPAS2 sponging of miR-7-5p up-regulates EGFR to activate the PI3K/AKT/c-MYC pathway (Zhang et al., 2020a). Human glioma cells have low amounts of lncRNA UBE2R2-AS1. Glioma cells growth is in part regulated by the UBE2R2-AS1/miR-877-3p/TLR4 pathway. Following upregulation of UBE2R2-AS1 in glioma cells, there was a remarkable elevation of caspase-3, caspase-7, and caspase-8, while reduction of Bcl2 (Xu et al., 2019c). LncRNA Gas5 is down-regulated in glioma. The Overexpression of Gas5 promotes the apoptosis. Gas5 and miR- 222 expressions are inversely associated. The overexpression of Gas5 upregulates Plexin C1, bmf by lowering the level of miR-222. The down-regulation of miR-222 increases the level of bmf (a pro-apoptotic agent), which increase Bax expression and decrease Bcl-2 expression in glioma (Zhao et al., 2015). lncRNA ZEB1-AS1 is elevated in glioma. Knockdown of ZEB1-AS1 induces apoptosis by increasing Bax and lowering Bcl-2 (Lv et al., 2016). LncRNA H19 expression was up-regulated in glioblastoma cells. H19 knockdown leads to increasing apoptosis in glioblastoma cells under TMZ treatment. H19 knockdown reduces pro-caspase 3 and increases cleaved caspase 3 and Bax, and decreases Bcl-2 in glioblastoma cells under TMZ treatment (Li et al., 2016). High levels of lncRNA SNHG6 are present in glioma cells. The knockdown of SNHG6 increases apoptosis. By regulating miR-101-3p expression, SNHG6 exerts its effect in glioma tumorigenesis (Meng et al., 2018). NEAT1 is downregulated in glioma. Elevating the level of NEAT1 in glioma cells can enhance apoptosis. NEAT1 exerts its tumor suppressor effects by down-regulating miR-92b and up-regulating DKK3 (Liu et al., 2020). LncRNA SNHG16 is elevated in glioma. SNHG16 inhibition leads to apoptosis *via* upregulating caspase-3 and Bax and, down-regulating Bcl-2, Bcl-xl and Mcl-1. In addition, SNHG16 may affect apoptosis via regulation of PI3K/Akt pathway. By binding to MiR-4518 and its inhibition, SNHG16 can regulate PRMT5 expression. SNHG16 could exerts oncogenic function through the SNHG16-miR-4518 axis in glioma cells (Lu et al., 2018). Apoptosis of glioma cells could be enhanced by inhibiting lncRNA EPIC1. The down regulation of EPIC1 reduces the Cdc20 expression which functions as an anti-apoptotic agent in glioma cells (Wang et al., 2020a). LncRNA SOX2OT is present in high levels in glioma. SOX2OT inhibition upregulates miR-194-5p and miR-122 which in turn enhance the apoptosis of glioblastoma stem cells (Su et al., 2017). Glioblastoma cells have low levels of LncRNA CASC2. LncRNA CASC2 acts as a tumor suppressor by stimulating apoptosis through CASC2-miR-18a axis in glioblastoma (Wang et al., 2020b). LncRNA GAPLINC was significantly up-regulated in glioblastoma tissues. GAPLINC inhibition promoted apoptosis. GAPLINC can target miR-331-3p. GAPLINC could be an oncogenic lncRNA via negative modulation of miR-331-3p in glioblastoma (Chen et al., 2019). The overexpression of LncRNA LINC00657 leads to miR-190a-3p inhibition; Consequently, PTEN expression is stimulated regulating caspase3 through PI3K/Akt/mTOR pathway. Thus, the overexpression of LncRNA LINC00657 induces apoptosis in glioblastoma (Chu et al., 2019). LncRNA SNHG20 was highly expressed in glioblastoma. The knockdown of SNHG20 significantly promotes cell apoptosis. PI3K/Akt/mTOR signaling pathway were inhibited by SNHG20 knockdown, while were stimulated by SNHG20 overexpression. Apoptosis is increased with IGF-1, a PI3K signaling activator, whereas apoptosis is decreased with GDC-094, a PI3K signaling inhibitor. Apoptosis induced by SNHG20 knockdown is efficiently rescued by IGF-1 (Gao et al., 2019b). Glioblastoma stem cells have high amounts of LncRNA NEAT1. LncRNA NEAT1 inhibition promotes glioblastoma stem cells apoptosis. There is a binding region between LncRNA NEAT1 and microRNA let-7e. NEAT1 inhibition increases the level of let-7e which can downregulate NRAS (a pro-oncogenic factor) as its target. In addition, the overexpression of NRAS significantly inhibit apoptosis in glioblastoma stem cells (Gong et al., 2016). LncRNA AGAP2-AS1 is elevated in glioblastoma and its inhibition leads to TFPI2 overexpression and enhanced apoptosis (Luo et al., 2019). LncRNA MANTN1-AS1 was down-regulated in glioblastoma. LncRNA MANTN1-AS1 induce glioblastoma cell apoptosis via regulation of different proteins RELA, ERK1/2, survivin, MMP-9, Bcl-2 and Bax (Han et al., 2019). LncRNA DLEU1 was significantly up-regulated in glioblastoma tissues. DLEU1 knockdown increase Bax and decrease Bcl-2. SP1 is a target of miR-4429 and DLEU1 sponged miR-4429 to induce SP1 expression. Therefore, SP1–DLEU1–miR-4429 pathway could regulate apoptosis in glioblastoma (Liu et al., 2019). LncRNA HOTAIRM1 inhibits apoptosis in glioblastoma through regulation of miR-873-5p/ZEB2 axis. By inactivating miR-873-5p, HOTAIRM1 upregulates ZEB2 (Lin et al., 2020). LncRNA MALAT1 has pivotal role in ZHX1 expression. MALAT1 induce ZHX1 expression through miR-199a in glioblastoma cells. The overexpression of ZHX1 is related to decreased Bax and increased Bcl-2 in glioblastoma (Liao et al., 2019a). In another study showed a significant up-regulation of LncRNA LEF1-AS1 expression in glioblastoma. The Knockdown of *LEF1-AS1* significantly promoted cell apoptosis through regulating p27, Blc-2, and Bax expression (Wang et al., 2017). LINC01152 was up-regulated in glioblastoma. The silenced LINC01152 decreases the levels of MAML2. The apoptosis of glioblastoma cells is stimulated by MAML2 depletion. LINC01152 and MAML2 3′UTR have binding sites for miR-466. miR-466 inhibition could in part reverse the enhanced apoptosis observed after LINC01152 silencing (Wu et al., 2021). High levels of LncRNA SNHG3 are observed in glioma. SNHG3 inhibition induces cell apoptosis (Fei et al., 2018). In glioma cells, the silencing of lncRNA PVT1 inhibits EZH2 which in turn increases caspase 3 and Bax and reduces Bcl-2 and as a result, apoptosis is promoted (Yang et al., 2017). LncRNA GATA6-AS was up-regulated in glioma. GATA6-AS overexpression results in TUG1 downregulation and apoptosis inhibition (Liao et al., 2019b). LncRNA UBA6-AS1 expression was enhanced in glioblastoma. Through miR-760/HOXA2 regulation, UBA6-AS1 inhibition can improve apoptosis (Cheng et al., 2021). LncRNA KCNQ1OT1 significantly was Up-Regulated in glioma. LncRNA KCNQ1OT1 down-regulation induces glioma cells apoptosis. KCNQ1OT1 expression inversely correlates with miR-370 expression in glioma cells. Apoptosis is increased by miR-370 upregulation. MiR-370 exerts its anti-oncogenic effects through CCNE2 downregulation (Gong et al., 2017). LncRNA SCAMP1 is observed in high levels in glioma cells. The inhibition of SCAMP1 promotes apoptosis via molecular sponging of miR-499a-5p (Zong et al., 2019). LncRNA HOTAIR expression was up-regulated in glioblastoma. There is positively correlation between HOTAIR with the HK2 expression. HK2 depletion sensitizes the glioblastoma cells to TMZ-induced apoptosis. HK2 absence in glioblastoma cells also enhances the cleavage of caspase-3 following TMZ treatment and induces higher apoptosis Depletion of HOTAIR also suppresses the expression of HK2 increases the TMZ-induced apoptosis and cleavage caspase-3 in glioblastoma cells. The expression of miR-125 up-regulates by HOTAIR knockdown and miR-125 could be the downstream of HOTAIR for HK2 regulation (Zhang et al., 2020b). Low levels of LncRNA HOTTIP is observed in glioma. HOTTIP upregulation increases cell apoptosis. HOTTIP directly binds to BREgene and down-regulate BRE expression. Through BRE downregulation which results in cyclin A and CDK2 suppression and P53 elevation, HOTTIP reduces glioma cell growth (Xu et al., 2016). LncRNA LINC00515 was over-expressed in glioma tissues. LINC00515 deficiency increases apoptosis in glioma cells. LINC00515 regulates PRMT5 expression *via* sponging miR-16 (Wu and Lin, 2019). A remarkably low level of LncRNA PART1 is observed in glioma. PART1 inactivates miR-190a-3p which suppresses PTEN/AKT axis and as a result apoptosis in enhanced in glioma cells (Jin et al., 2020a). LncRNA WEE2-AS1 is elevated in glioblastoma. Through miR-520f-3p/SP1 axis, WEE2-AS1 inhibition improves apoptosis in glioblastoma (Lin et al., 2021). LncRNA Linc-00313 is elevated in glioma. Linc-00313 suppression significantly promotes apoptosis in glioma; this effect is due to miR-342-3p and miR-485- 5p upregulation (Shao et al., 2019). LncRNA AC003092.1 is lowered in glioblastoma. LncRNA AC003092.1 exerts its effects in TMZ chemosensitivity by regulating miR-195/TFPI-2 pathway (Xu et al., 2018a). Glioma cells have elevated levels of lncRNA SNHG12. The overexpression of lncRNA SNHG12 significantly inhibit the apoptosis in glioma cell *via* targeting Hu antigen R (HuR) (Lei et al., 2018). LncRNA HOTAIRM1 is upregulated in glioma and glioblastoma. HOTAIRM1 inhibited cell apoptosis via regulation of HOXA1 gene (Li et al., 2018). The overexpression of lncRNA PABPC1 increased apoptosis in glioblastoma cells via BDNF-AS-RAX2-DLG5 axis (Su et al., 2020). Glioblastoma cells show elevated levels of LncRNA PXN-AS1. Apoptosis of glioblastoma cells was promoted by PXN-AS1 inhibition through lowering Bcl-2 and elevating Bax (Chen et al., 2020). There are low levels of TAF15 and LncRNA LINC00665 in glioma, on the contrary, high levels of MTF1, YY2, and GTSE1 are observed. TAF15 upregulation could promotes apoptosis *via* targeting MTF1,YY2, and GTSE1 (Ruan et al., 2020). LncRNA MATN1-AS1 is lowered in glioblastoma. MATN1-AS1 upregulation is able to promote apoptosis through RELA, survivin, ERK1/2, MMP-9, and Bcl-2 inhibition, and Bax elevation (Han et al., 2019).

Medulloblastoma tissues are observed to have elevated levels of LncRNA HOTAIR which binds to miR-1 and miR-206 and inhibits them to upregulate YY1. Through miR-1/miR-206-YY1 pathway, HOTAIR suppression leads to increased apoptosis in medulloblastoma (Zhang et al., 2020c). Medulloblastoma tissues demonstrate high levels of LncRNA LOXL1-AS1. The proliferation and metastasis of medulloblastoma cells are improved by LOXL1-AS1 *via* stimulating PI3K-AKT pathway and therefore, its suppression results in increased apoptosis (Gao et al., 2018). There is an elevated level of LncRNA TP73-AS1 in medulloblastoma cells which binds to miR-494-3p to inhibit it and in turn activate EIF5A2, the pathway that explains promoted apoptosis following TP73-AS1 suppression (Li et al., 2019b). LncRNA CRNDE was up-regulated in medulloblastoma. The knockdown of lncRNA CRNDE induces apoptosis via promoting the activity of caspase-3 in medulloblastoma cells (Song et al., 2016) Cleaved-caspase-3, caspase-9, and Bax elevation, and bcl-2 reduction are the results of HOTAIR downregulation in medulloblastoma cells (Zhao et al., 2020). Varon et al. (2019) reported a significant up-regulation of lncRNA TP73-AS1 in medulloblastoma cells. Silencing TP73-AS1 stimulates apoptosis via targeting caspase 3 in medulloblastoma cells.

Malignant meningioma shows LncRNA LINC00702 to be upregulated. LINC00702 stimulates tumorigenesis by inactivating miR-4652-3p which consequently activates ZEB1. Therefore, LINC00702 downregulation induces apoptosis (Li et al., 2019c). Astrocytoma cells are shown to have high amounts of LncRNA SNHG17. SNHG17 can bind to miR-876-5p to keep it from sponging ERLIN2. This pathway justifies the enhanced apoptosis observed after SNHG17 inhibition (Du and Hou, 2020). Table 2 lists apoptosis-related lncRNAs in brain tumors and their expression.

**TABLE 2:** Apoptosis-related lncRNAs in brain tumors.

	Genomic coordinates	Brain tumors	lncRNA	Targets	Model	Type of cell line	Ref
Up regulated lncRNAs expression	p36.32 chr1:3735510–3745905 (-)	Medulloblastoma	TP73-AS1	miR-494-3p/EIF5A2	*In vivo*	specimens of medulloblastoma human medulloblastoma cell lines Daoy, D341 animal experiment	Li et al. (2019b)
				E-cadherin, N-cadherin, Vimentin	*In vitro*		
	p36.32 chr1:3735510–3745905 (-)	Medulloblastoma	TP73-AS1	Capase-3	*In vivo*	cell lines UW228.2, MED8A and ONS76, DAOY/mice	Varon et al. (2019)
					*In vitro*		
	p35.3 chr1:28505979–28510892 (+)	Glioblastoma	SNHG3		Human, *in vitro*	glioma tissue/cell lines (A172, U251, U87, and SHG44)	Fei et al. (2018)
	p35.3 chr1:28578537–28581010 (-)	Glioblastoma	SNHG12	HuR	*In vitro*	tumor samples U87, LN229, U373, U251	Lei et al. (2018)
	q27.3 chr3:188151205–188154057 (-)	Glioblastoma	LPP-AS2	miR-7-5p/EGFR/PI3K/AKT/and c-MYC PI3K/AKT pathway	*In vitro*	Glioma tissues U251, U87, SHG44, T98G, GOS-3, TJ905, U373	Zhang et al. (2020a)
					*In vivo*		
	q26.33 chr3:180989761–181117494 (+)	Glioblastoma	SOX2-OT	MiR-194-5p, miR-122	*In vivo*	U87 and U251 cell lines	Su et al. (2017)
	q13.2 chr4:67701208–67723914 (+)	Glioblastoma	UBA6-AS1	miR-760/HOXA2	*In vivo*	GBM tissues/GBM cell lines A172 and U251	Cheng et al. (2021)
					*In vitro*		
	q25 chr4:108167524–108176426 (+)	Glioblastoma	LEF1-AS1	ERK1/2 and Akt/mTOR pathway	*In vivo*	GBM tissues Human U251 and U87 glioma cells	Wang et al. (2017)
				Bax and Bcl-2	*In vitro*		
	q14.1 chr5:78360610–78476026 (+)	Glioblastoma	SCAMP1	Wnt/β‐catenin	*In vivo*	Glioma specimens U87, U251, HEK293T mice	Zong et al. (2019)
	p15.2 chr7:27184506–27189298 (+)	Glioblastoma	HOXA11-AS	miR-130a-5p/HMGB2	*In vitro*	Glioma tissues and U251 and U87MG cells	Xu et al. (2019b)
					*In vivo*		
	p11.2 chr7:55179749–55188934 (-)	Glioblastoma	EGFR-AS1	miR-133b/RACK1	*In vitro*	Human U87, U251 and T Human glioma specimens98 G	Dong et al. (2019)
				N-cadherin, Vimentin and MMP-2, Bax, cleaved Caspase-3 and PARP	*In vivo*		
	q34 chr7:141704002–141738230 (-)	Glioblastoma	WEE2-AS1	miR-520f-3p/SP1	*In vivo*	GBM tissues/GBM cell lines T98 and U138/mice	Lin et al. (2021)
					*In vitro*		
	p15.2 chr7:27095646–27096327 (+)	Glioblastoma	HOTAIRM1	HOXA1	*In vivo*	U87, U251, and A172 cell lines glioma tissues	Li et al. (2018)
					*In vitro*		
	p15.2 chr7:27095646–27096327 (+)	Glioblastoma	HOTAIRM1	miR-873-5p/ZEB2	*In vitro*	U87, LN-229, U-251, and A172	Lin et al. (2020)
				Cyclin A1, Cyclin D1, Bcl-2, Caspase-3			
	q13.1 chr8:66921683–66925541 (-)	Glioblastoma	SNHG6	miR-876-5p/ERLIN2 axis	*In vitro*	LN-215, ADF, U138, A-382	Du and Hou, (2020)
	q24.21 chr8:127794525–128101256 (+)	Glioblastoma	PVT1	Bax/bcl2/caspase 3	*In vivo*	glioma cell lines U87MG and U251/glioma tissue/mice	Yang et al. (2017)
					*In vitro*		
	p11.22 chr10:31206277–31319691 (-)	Glioblastoma	ZEB1-AS1	Bax/bcl2	*In vivo in vitro*	HS683, T98G, U87, U251 glioma tissues	Lv et al. (2016)
	p15.1 chr10:4201140–4243912 (-)	Meningioma	LINC00702	miR-876-5p/ERLIN2 axis	*In vitro*	LN-215, ADF, U138, A-382	Du and Hou, (2020)
	q13.1 chr11:65497687–65506431 (+)	Glioblastoma	MALAT1	miR-199a/ZHX1	*In vivo*	Glioma tissues	Liao et al. (2019a)
				Bcl-2/Bax	*In vitro*	U87-MG, U251, T98G, and A172	
	q13.1 chr11:65422773–65426457 (+)	Glioblastoma	NEAT1	miRNA let-7e/NRAS	Human, *In vitro*	glioma tissues	Gong et al. (2016)
						U87, T98G, U251, A272 and U373 cell lines	
	p15.5 chr11:1995175–1996191 (-)	Glioblastoma	H19	Notch signaling pathway	*In vivo*	GBM tissue GBM cell lines (M059K, LN-229, T98G, and U343) mice	Wu et al. (2021)
					*In vitro*		
	p15.5 chr11:2608327–2699994 (-)	Glioblastoma	KCNQ1OT1	miR-370/CCNE2	*In vitro*	cell lines (U87, U251)/glioma tissues/mice	Gong et al. (2017)
					*In vivo*		
	q24.23 chr12:120201290–120213231 (+)	Glioblastoma	PXN-AS1	Wnt/β-catenin pathway	*In vivo*	A172, U251, U87, LN229	Chen et al. (2020)
						Animal sample	
	q13.13 chr12:53962307–53974956 (-)	Medulloblastoma	HOTAIR	miR-1/miR-206/YY1	*In vivo*	primary medulloblastoma samples	Zhang et al. (2020c)
						Human cells, Daoy, D283 med and D34	
	q13.13 chr12:53962307–53974956 (-)	Medulloblastoma	HOTAIR	caspase-3,9/bcl2/bax	*In vivo*	medulloblastoma cell lines Daoy and D341/mice	Zhao et al. (2020)
	q13.13 chr12:53962307–53974956 (-)	Glioblastoma	HOTAIR	Caspase-3	*In vivo*	U-87 and A172 cell lines/GBM samples/mice	Zhang et al. (2020b)
					*In vitro*		
	q14.1 chr12:57726270–57728356 (+)	Glioblastoma	AGAP2-AS1	TFPI2	*In vivo*	GBM tumor tissues	Luo et al., 2019)
					*In vitro*	GBM cell lines (A172, U87/MG, U251/MG, LN229, and SHG44)	
	q14.2 chr13:50082168–50107202 (+)	Glioblastoma	DLEU1	miR-4429	?	U251, U87 and LN229	Liu et al. (2019)
				Bcl-2/Bax/caspase-3			
	q24.1 chr15:73908070–73919870 (-)	Medulloblastoma	LOXL1-AS1	PI3K-AKT	*In vivo*	Human tissues	Gao et al. (2018)
					*In vitro*	Human medulloblastoma cell lines cell lines Daoy, D283, D425, D341, and D458	
	q12.2 chr16:54845188–54848278 (-)	Medulloblastoma	CRNDE	miR-101-3p	*In vivo*	glioma tissues U87, U251, LN229, T98G Mice	Meng et al. (2018)
	q24.3 chr17:72030290–72036316 (+)	Glioblastoma	LINC01152	caspase-3	*In vivo*	cell lines D283, Daoy, D425, D341, and D458/Human Samples/Mouse	Song et al. (2016)
					*In vitro*		
	q25.1 chr17:76557763–76564689 (+)	Glioblastoma	SNHG16	Bcl/bax/caspase-3/PI3K/Akt	*In vitro*	brain glioma specimens/U251, H4, SW1783 and LN229 cells/	Lu et al. (2018)
	q25.2 chr17:77086715–77093762 (+)	Glioblastoma	SNHG20	PI3K/Akt/mTOR	*in vivo*	glioblastoma tissues glioblastoma cell lines (U87MG, U343, U251, LN215)	Gao et al. (2019b)
	p11.31 chr18:3466249–3473203 (+)	Glioblastoma	GAPLINC	miR-331-3p	*in vitro*	GBM cell lines (T98G, U251, LN18, LN229, and A172)	Chen et al. (2019)
					*in vivo*		
	q11.2 chr18:22164885–22167781 (-)	Glioblastoma	GATA6-AS1	TUG1	*In vitro*	Tumor tissues glioma cell lines Hs 683 and CCD-25Lu	Liao et al. (2019b)
	q11.23 chr20:38419637–38435375 (-)	Astrocytoma	SNHG17	Caspase 3/bcl2/bax	*In vitro*	cell lines U87MG, U251, U343, Hs683, LN215 and A172	Li et al. (2016)
	q21.3 chr21:25582769–25583326 (-)	Glioblastoma	LINC00515	miR-16/PRMT5	*In vivo*	Glioma tissue/Human glioma cell lines U251, U87MG, LN229	Wu and Lin, (2019)
	q22.3 chr21:4,3461959–43478223 (-)	Glioblastoma	Linc-00313	UPF1-Linc-00313-miR-342-3p/miR-485-5p-Zic4-SHCBP1	*In vivo*	(GBM) cell lines U87, U251/glioma tissues/mice	Shao et al. (2019)
	q13.31 chr22:47630827–48023030 (+)	Globlastoma	EPIC1 (LOC284930)	Notch signaling pathway	*In vivo*	GBM tissue GBM cell lines (M059K, LN-229, T98G, and U343) mice	Wu et al. (2021)
					*In vitro*		
Down regulated lncRNAs expression	p35.2 chr1:30718503–30726744 (+)	Glioblastoma	MATN1-AS1	RELA	*in vivo in vitro*	U87MG and U251	Han et al. (2019)
				ERK1/2, Bcl-2, survivin, andMMP-9, Bax		GBM tissue	
	p35.2 chr1:30718503–30726744 (+)	Glioblastoma	MATN1-AS1	Bcl/bax	*In vivo*	GBM cells U87MG and U251/GBM tissue specimens/mice	Han et al. (2019)
					*In vitro*		
	q25.1 chr1:173858558–173867045 (-)	Glioblastoma	GAS5	Bcl-2\bax	*In vivo*	cell lines (U87 and U251)/glioma tissues/mice	Zhao et al. (2015)
					*In vitro*		
	q12.1 chr5:60487712–60491518 (+)	Glioblastoma	PART1	PI3K/AKT	*In vitro*, human	U87MG, LN-18, LN-428 glioma tissues	Jin et al. (2020a)
				Bcl2/bax			
	p15.2 chr7:27198574–27201236 (+)	Glioblastoma	HOTTIP	P53	*In vivo in vitro*	A172, U251, U87-MG, U118-MG glioma tissues mice	Xu et al. (2016)
	q21.3 chr7:94022832–94064723 (+)	Glioblastoma	AC003092.1	miR-195/TFPI-2 signaling	*In vivo*	Human U87 cell line/glioma tissues/mice	Xu et al. (2018a)
					*In vitro*		
	p13.3 chr9:33775182–33801186 (-)	Glioblastoma	UBE2R2-AS1	miR-877-3p/TLR4	*In vitro*	U251, A-172, U87-MG, and U373 glioma samples	Xu et al. (2019c)
				caspase-3/-7/-8 and Bcl2	*In silico*		
	q26.11 chr10:118046278–118206659 (+)	Glioblastoma	CASC2	miRNA-18a	*In vitro*	glioblatoma tissue	Wang et al. (2020b)
				E-cadherin	*In vivo*	Human glioblastoma cells (A172 and T98)	
	p14.1 chr11:27506829–27515218 (+)	Glioblastoma	BDNF-AS	PABPC1-BDNF- AS-RAX2-DLG5 pathway	*In vivo*	U87 and U251 Glioma tissues Mice	Su et al. (2020)
	q13.1 chr11:65422773–65426457 (+)	Glioblastoma	NEAT1	miR-92b/DKK3 pathway		U-87 MG and U251 glioma tissues	Liu et al. (2020)
	q13.12 chr19:36259539–36264788 (-)	Glioblastoma	LINC00665	TAF15/LINC00665/MTF1(YY2)/GTSE1 axis	*In vivo*	U251 and U87 glioma cells/glioma tissues/mice	Ruan et al. (2020)
					*In vitro*		
	q11.23 chr20:36045617–36051018 (-)	Glioblastoma	LINC00657 (NORAD)	miR-190a-3p/PTEN	*In vivo*	U-87 MG, LN-18 and U-118 MG	Chu et al. (2019)
				PI3K/Akt/mTOR	*In vitro*	Animal cases

### Biogenesis of the Circular RNAs

Circular RNAs (circRNAs) characterize a type of endogenous ncRNA generated by back splicing events, with the ubiquitous presence in numerous species. They have one or more exons with the major location in the cytoplasm so that a few of the circRNAs consisting of intron originate in the nucleus (Guo et al., 2014). In spite of the linear RNAs, circRNAs make a continuous loop formation using covalent bonds and therefore do not have a 5–3′ direction or a polyadenylated tail; these features result in their higher stability in the plasma and tissues (Suzuki and Tsukahara, 2014).

Hence, the mentioned specific structural features imply the fact that they are pathologically and physiologically major transcripts. A lot of investigations have confirmed that the abnormal expression of circRNAs is one of the major regulatory elements in expansion and carcinogenesis of some cancers such as hematological malignancies, lung cancer, and liver cancer, revealing the probable targets to diagnosis and prognosis of these cancers by circRNAs. Some recent investigations performed about features and biogenesis of the circRNAs, summarized their functions and mechanisms of action in cancers, and discussed their capacity to diagnose and treat the diseases of interest (Bach et al., 2019; Zhang et al., 2020d; Lei et al., 2020; Shen et al., 2021).

**FIGURE 4:**
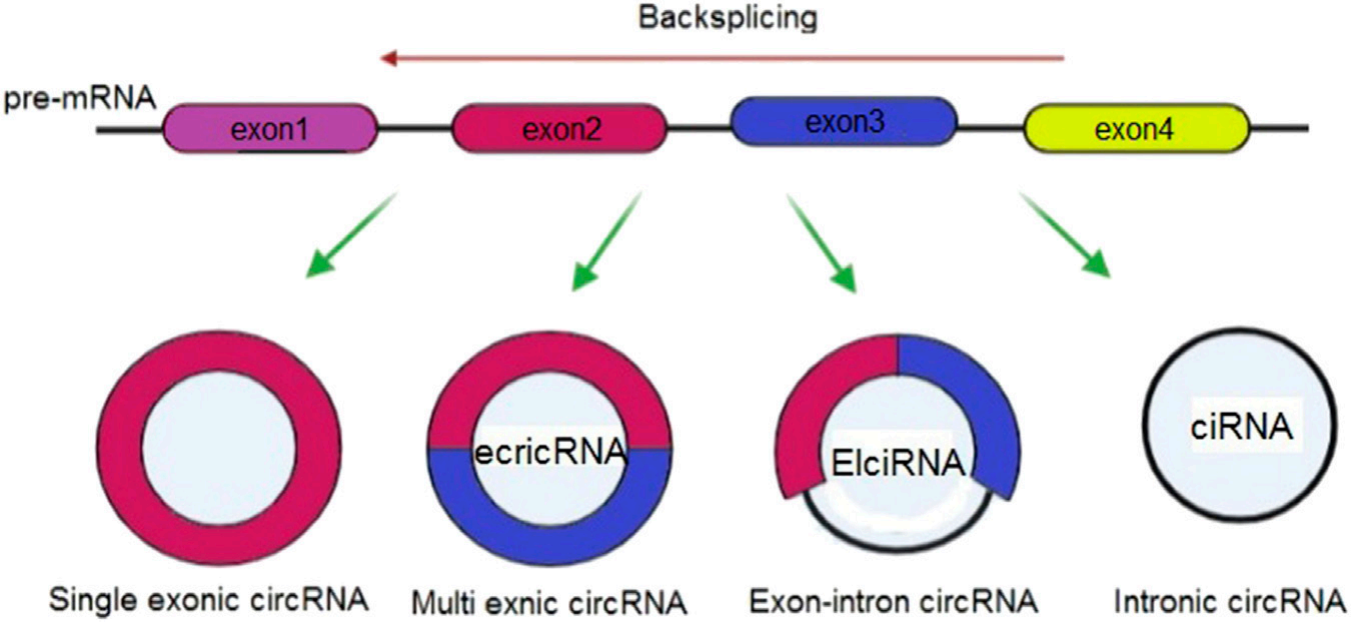
Classification of circular RNAs.

Considering their origins, it is possible to classify circRNAs into 3 groups of circular intron RNAs (ciRNAs), exonic circRNAs (ecircRNAs), and exon–intron circRNAs (EIciRNAs) (Meng et al., 2017) (Figure 4). Various circularization mechanisms regulate circRNA biogenesis, out of which ecircRNA is abundantly found so that it includes most of specified circRNAs (Bolha et al., 2017). In fact, gene transcription is regulated by cirRNAs. Overall, Pol II transcribes a pre-mRNA which consists of exons and introns as well as a seven- methylguanosine cap and poly-adenosine tail, which is added to its 5′- and 3′-ends. After that, pre-mRNA is spliced at the canonical splice sites (5′-GU & 3′-AG at introns) using spliceosomes for maturity and translatability. In addition, one of the specific splicing manners known as back-splicing generates CircRNA, where the 3′-end of an exon binds to the 5′-end of its own or an upstream exon via a 3′, 5′- phosphodiester bond, and establishes a closed structure with a back-splicing junction. Researchers viewed circRNAs as the splicing errors consisting of scrambled exons, and presented and verified two biogenesis models based on the splicing events sequence and various intermediates, including direct backsplicing model and lariat model (Zhang et al., 2020d; Zhou et al., 2020).

### Circular RNAs and Apoptosis in Brain Tumors

Glioblastoma cells show a remarkably high level of Circular RNA hsa_circ_0067934. The hsa_circ_0067934 silencing promotes apoptosis in glioblastoma cells via PI3K-AKT pathway (Xin et al., 2019). MiR‐181a was down-regulated in glioma, while the circ_0076248 and SIRT1 were up-regulated. The down-regulation of hsa_circ_0076248 or miR-181a overexpression upregulate p53 and SIRT1. Down regulation of hsa_circ_0076248 leads to inhibiting xenograft tumors’ growth and increasing apoptosis (Lei et al., 2019). CircPVT1 was over-expressed in glioblastoma tissues. Silencing circPVT1 increasese the number of apoptotic cells by increased cleaved-Caspase-3 and cleavedCaspase-9 expression via targeting miR-199a-5p (Chi et al., 2020). Glioblastoma cells were shown to have a greatly elevated level of circNT5E. circNT5E up-regulation inhibits apoptosis *via* miR-422a (Wang et al., 2018a). Glioma cells have a high level of Circ-TTBK2 and EZH2. Through miR-520b/EZH2 pathway, circ-TTBK2 suppression is able to improve apoptosis (Yuan et al., 2019). The high level of circSMO742 and SMO can inhibit miR-338-3p which results in increased proliferation, migration and invasion and decreased apoptosis of glioma cells (Xiong et al., 2019). Cir-ITCH is observed in low amounts in glioma. Through miR-214 inactivation and modulation of ITCH-Wnt/*β*-catenin axis in glioma cells, cir-ITCH upregulation can improve apoptosis (Wang et al., 2018b). Glioma cells have an elevated level of circPOSTN. *Via* targeting miR-361-5p/TPX2, circPOSTN suppression can lower Bcl-2 and elevate Bax cand caspase-3 which result in apoptosis (Long et al., 2020). CircPIP5K1A was up-regulated in glioma cells. The apoptosis increases after the knockdown of circPIP5K1A through TCF12/PI3K/AKT axis regulation by miR-515-5p inactivation (Zheng et al., 2021). Circ-MAPK4 was over-expressed in glioma tissues. Circ-MAPK4 silencing increases apoptosis by elevating caspase-3, caspase-7, caspase-9, and PARP1 via targeting miR-125a-3p and regulating p38/MAPK (Peng et al., 2020). Glioma cells show a greatly high level of circ-TTBK2. circ-TTBK2 regulates miR-217/HNF1β/Derlin-1 pathway which reduces apoptosis (Zheng et al., 2017). Glioma cells show hsa_circ_0088732 to be upregulated. hsa_circ_0088732 inhibition stimulates apoptosis through the miR-661/RAB3D pathway (Jin et al., 2020b). Glioma cells show circNFIX to be overexpressed. circNFIX impedes miR-34a-5p which inhibits apoptosis and therefore, circNFIX knockout can show the opposite effects (Xu et al., 2018b). CircKIF4A expression was increased in glioma. By targeting miR-139-3p which downregulates cyclin D1, c-Myc and Bcl2, and upregulates Bax, CircKIF4A suppression can improve apoptosis in glioma cells (Huo et al., 2020). Glioma cells show circPRKCI to be overexpressed. CircPRKCI shRNA could induce apoptosis by increasing Annexin V, and cleaving caspase-3, caspase-9 and PARP via targeting miR-545 (Zhang et al., 2019b). circNFIX is present in high amounts in glioma tissues. circNFIX suppression targets miR-378e/RPN2 pathway to induce apoptosis (Ding et al., 2019). hsa_circ_00037251 inhibition promotes apoptosis in glioma *via* miR-1229-3p/mTOR pathway (Cao et al., 2019). Radioresistant glioma was shown to have notably elevated levels of circ_VCAN. It exerts a carcinogenic role in glioma via regulating microRNA-1183. The knockdown of circ_VCAN stimulated apoptosis in glioma cells (Zhu et al., 2020). CircSKA3 was up-regulated in medulloblastoma tissues. sicircSKA3 reduces Bcl-2, while elevates caspase-3 in medulloblastoma cells by targeting miR-383-5p (Wang et al., 2020c). Table 3 illustrates apoptosis-related circular RNAs in brain tumors and their expression.

**TABLE 3:** Apoptosis-related circular RNAs in brain tumors.

	Genomic coordinate	Brain tumors	Circular RNAs	CircbaseID	Targets	Model	Type of cell line	Ref
Up regulated circular RNA expression	q21.3 chr1:151206672–151212515 (+)	Glioblastoma	CircPIP5K1A	hsa_circ_0014130	PI3K/AKT	*In vivo*	U87, TJ861, TJ905, U251, H4, A172 glioma tissues	Zheng et al. (2021)
						*In vitro*	mice	
	q26.2 chr3:170013698–170015181 (+)	Glioblastoma	CircPRKCI	hsa_circ_0067934	PI3K-AKT pathway	*In vitro*	GBM samples	Xin et al. (2019)
							The LN18, U251, LN229, T98G and A172 cells	
	q26.2 chr3:170013698–170015181 (+)	Glioblastoma	CircPRKCI	hsa_circ_0067934	caspase-3, caspase-9 and PARP	*In vivo*	A172 glioma cells/human glioma tissues/mice	Zhang et al. (2019b)
						*In vitro*		
	q14.2 chr5:82832825–82838087 (+)	glioblastoma	CircVCAN	hsa_circ_0073237	microRNA-1183	*In vitro*	The 293T, U87, and U251 cells glioma tissues/mice	Zhu et al. (2020)
	p21.2 chr6:37787306–38084515 (+)	Glioblastoma	CircZFAND3	hsa_circ_0076248	MiR‐181a/SIRT1	*In vivo*	Human GBM samples/U251, U87 cell lines	Lei et al. (2019)
						*In vitro*	Animal studies	
	q14.3 chr6:86176777–86197207 (+)	Glioblastoma	CircNT5E	hsa_circ_0077231	NT5E, SOX4, PI3KCA, p-Akt, p-Smad2	*In vivo*	Tumor tissue samples/GBM cells (U87 and U251)/mice	Wang et al. (2018a)
						*In vitro*		
	q32.1 chr7:128845043–128846428 (+)	Glioblastoma	CircSMO742	hsa_circ_0001742	miR-338-3p/SMO	*In vivo*	10 human gliomas tissues/human glioblastoma A172 and U-87 MG/mice	Xiong et al. (2019)
						*In vitro*		
	q24.21 chr8:128806778–128903244 (+)	Glioblastoma	CircPVT1	hsa_circ_0085536	miR-199a-5p	*In vitro*	human GBM tissues U539 and U251 cells	Chi et al. (2020)
					YAP1 and PI3K/AKT			
					Capase-3			
					Capase-9			
					Ncadherin, Vimentin, Zeb1, E-cadherin			
	q34.12 chr9:130914461–130915734 (+)	Glioblastoma	CircLCN2	hsa_circ_0088732	N-cadherin, vimentin, E-cadherin	*In vivo*	tumor tissues/glioma cell lines	Jin et al. (2020b)
						*In vitro*	LN229, U87-MG, U251, and A172/mice	
	q12.11 chr13:21735928–21746820 (-)	Medulloblastoma	CircSKA3	hsa_circ_0029696	miR-383-5p/FOXM1	*In vivo*	tissue samples/cell lines	Wang et al. (2020c)
					Bcl2 and capase-3	*In vitro*	DAOY and ONS-76	
	q13.3 chr13:38136718–38161065 (-)	Glioblastoma	CircPOSTN	hsa_circ_0030018	Bcl2/bax/caspase 3	*In vivo*	Human tissue sample/Glioma cell line (U251, LN229)/mice	Long et al. (2020)
						*In vitro*		
	q15.2 chr15:43120125–43164956 (-)	Glioblastoma	CircTTBK2	hsa_circ_0000594	Mirna-520b/EZH2	*In vivo*	glioma tissues	Yuan et al. (2019)
						*In vitro*	A172 and U251 mice	
	q15.2 chr15:43120125–43164956 (-)	Glioblastoma	CircTTBK2	hsa_circ_0000594	PI3K/AKT and ERK	*In vivo*	Human tissues specimens/Human U87 and U251 glioma cell lines mice/	Zheng et al. (2017)
						*In vitro*		
	p13.3 chr16:765172–767480 (+)	Glioblastoma	CircMETRN	hsa_circ_0037251	miR-1229-3p/mTOR axis	*In vivo*	U373, U251 mice	Cao et al. (2019)
						*In vitro*		
	q21.1 chr18:48189458–48190874 (+)	Glioblastoma	CircMAPK4	hsa_circ_0047688	p38/MAPK	*In vitro in vivo*	U138, U373 and U87 glioma cell lines/glioma tissues/mice	He et al. (2020)
	p13.13 chr19:13183860–13192669 (+)	Glioblastoma	CircNFIX	hsa_circ_0049658	Notch signaling pathway	*In vivo*	SF-539, SHG-44	Xu et al. (2018b)
						*In vitro*	U87 glioma tissue from mice	
	p13.13 chr19:13183860–13192669 (+)	Glioblastoma	CircNFIX	hsa_circ_0049658	miR-378e/RPN2 axis	*In vivo*	Patient samples T98, U251, SW1783,A172	Ding et al. (2019)
						*In vitro*	Mice	
	q13.1 chrX:69606467–69607147 (+)	Glioblastoma	CircKIF4A	hsa_circ_0090956	Wnt5a, β-catenin, c-Myc, cyclin D1/bcl2/bax	*In vivo*	glioma sample LN229, A172, SHG44, U251s mice	Huo et al. (2020)
Down regulated Circular RNA expression	q11.21 chr20:33001547–33037285 (+)	Glioblastoma	CircITCH	hsa_circ_0001141	ITCH-Wnt/*β*-catenin	*In vitro*, human	cancer tissues U87, U251, A172, SHG44, LN229, T98G, SHG139	Wang et al. (2018b)

## Conclusion

Various cellular functions reveal the contribution of ncRNAs to the cancer development at the transcriptional, translational, as well as epigenetic, level. Hence, ncRNAs can be a hopeful path of molecular medicine, though, patterns of RNAs expression in the brain tumors are not constant and stable, and thus researchers must provide proper alternatives to be utilized as the diagnostic or treatment instruments. With their stable structure, various ncRNAs have shown their capacity to function as regulatory agents. Studies illustrated a few circRNAs, lncRNAs even snoRNAs as the potent treatment, diagnostic, prognostic targets in the tumors of brain. Therefore, there are limited investigations revealing the strict correlation of RNAs with prognosis of the cases with gliomas, WHO grade, as well as their real potential diagnostic value. Nonetheless, multiple investigations largely emphasized the pathological- clinical specimens. A major difficulty is that we must be wait for those research that specify circRNAs, snoRNAs, miRNA, and lncRNAs from the bodily fluids, in particular, CSF and blood of the cases with brain tumors. Hence, it is important to focus on the particular methods about stability, abundance, and longer half-life for identifying ncRNAs molecules associated with the noninvasive diagnosis and classification of the sub-types of gliomas. Hence, such attempts may simplify the evaluation of the initial treatment with higher sensitivity, magnetic resonance imaging (MRI), and or other conventional bio-markers. Thus, expression profile of the regulator RNAs that is identified by analyzing circRNAs, snoRNAs, miRNA, and lncRNA along with studying the respective effects would be of high importance for exploring the molecular pathology of the tumors in the brain. Finally, more research must be performed for developing one of the new RNA-based approaches for treating any malignant tumor and using their diagnostic and prognostic potency, which may result in promising results for cases with the brain tumors.
